# Promiscuous Expression of α-Tubulin II in Maturing Male and Female *Plasmodium falciparum* Gametocytes

**DOI:** 10.1371/journal.pone.0014470

**Published:** 2010-12-30

**Authors:** Samana Schwank, Colin J. Sutherland, Chris J. Drakeley

**Affiliations:** Department of Immunology and Infection, Faculty of Infectious and Tropical Diseases, London School of Hygiene and Tropical Medicine, London, United Kingdom; Agency for Science, Technology and Research (A*STAR), Singapore

## Abstract

**Background:**

Antimalarial interventions designed to impact on the transmissible sexual stages of *Plasmodium falciparum* are evaluated by measurement of peripheral gametocyte carriage *in vivo* and infectivity to mosquitoes. Drug or vaccine-elicited effects may differentially affect the relative abundance of mature male and female sexual forms, and this can be measured by estimation of sex ratios before and after intervention *in vivo* and *in vitro*. Measuring the impact of anti-gametocyte drugs on sexual commitment of immature gametocyte stages *in vitro* is not currently possible as male and female parasites cannot be distinguished by morphology alone prior to stage IV.

**Methodology/Principal Findings:**

We have modified an existing immunofluorescence-based approach for distinguishing male and female gametocytes during development *in vitro*, by using highly synchronised magnetically-enriched gametocyte preparations at different stages of maturity. Antibodies recognising α-tubulin II (males) and Pfg377 (females) were used to attempt to discriminate the sexes. Transcription of these two proteins was not coordinated during *in vitro* development, with *pfg377* transcripts accumulating only late in development, immediately prior to immunofluorescent signals from the PfG377 protein appearing in stage IV gametocytes. Contrary to previous descriptions of this protein as male-specific in *P*. *falciparum*, α-tubulin II recognised both male and female gametocytes at stages I to IV, but evidence of differential expression levels of this protein in late stage male and female gametocytes was found. Using antibodies recognising PfG377 as the primary marker and α-tubulin II as a secondary marker, robust estimates of sex ratio in *in vitro* cultures were obtained for gametocytes at stage IV or later, and validated by light microscopic counts. However, sex ratio estimation was not possible for early stage gametocytes due to the promiscuity of α-tubulin II protein expression, and the relatively late accumulation of PfG377 during the development process.

**Conclusions/Significance:**

This approach is a feasible method for the evaluation of drug impacts on late-stage gametocyte sex ratio in *in vitro* studies. Additional sex-specific antigens need to be evaluated for sex ratio estimation in early stage gametocyte preparations.

## Introduction

The propagation of malaria is a public health threat throughout the tropics. Recent calls for intensification of the effort towards malaria elimination have emphasised the need for drugs and vaccines that target gametocytes, particularly those of *P*. *falciparum*, in order to bring malaria transmission under control [1]. Recent renewed interest in the evaluation of the anti-gametocyte effects of therapeutic agents, both *in vitro* and *in vivo*, is thus very welcome [2], [3]. These evaluations require establishment of novel methods for studying gametocytes *in vitro*. One potential impact of interest is perturbation of the natural sex ratio, and recent studies have shown that changes in the proportion of male gametocytes taken up by feeding mosquitoes can affect transmission [4]. Rodent parasite studies strongly implicate sex ratio as an indicator of fitness in *P*. *chabaudi* [5]. Studies of *P*. *falciparum in vivo* have suggested that drug treatment may differentially affect the half-life of male and female gametocytes [6], and therefore may affect the transmission success of the parasite.

Currently, the standard method for quantifying the *P*. *falciparum* gametocyte sex ratio remains the identification of male and female gametocytes by light microscopy, using five discriminatory morphological characters [7]. Individual *P*. *falciparum* gametocytes from *in vitro* cultures have been sexed with alternative methods at low densities, including electron microscopy [8], *in situ* hybridization [9], immunoelectron microscopy [10] and immunofluorescent antibody test (IFAT) [11]–[16]. However, these methods are laborious, and hitherto have only been applicable to small numbers of specially prepared gametocytes, and have thus not been used to derive reliable estimates of the gametocyte sex ratio *in vitro*. Further, all studies to date have estimated sex ratios in preparations of mature stageIV/stageV gametocytes only, as sex-distinguishing morphological characteristics are absent in immature stages.

Discrimination between the gametocyte sexes by IFAT has relied on two relatively well characterised sexual stage proteins, Pfg377 (PFL2405c in the 3D7 genome database) used to identify female gametocytes and α-tubulin II (PFD1050w) which has been used to identify male gametocytes [13], [14]. α-tubulin II has been described as a gametocyte-specific, male-specific protein in *P*. *falciparum* [12]–[14], [17], [18]. However recent studies in rodent malaria parasites have demonstrated transcription and expression of the *P*. *berghei* homologue of this protein in both asexual parasites and female gametocytes in infected mice [19], [20]. In *P*. *falciparum*, there is some *in vitro* evidence of expression of the α-tubulin II protein in asexual parasites [21], although these authors analysed expression of this protein from bulk cultures and so could not rule out the presence of some young parasites committed to sexual development. However, Khan *et al*. [19] were able to use α-tubulin II in transfected *P*. *berghei* parasites as a marker to separate male from female gametocytes using fluorescent flow cytometry, suggesting much higher expression levels are found in male compared to female gametocytes, at least in this rodent parasite. The utility of this protein as a potential male-specific probe in *P*. *falciparum* thus remains unclear.

A strategy for discriminating *P*. *falciparum* gametocyte sexes based on differential antibody staining by IFAT was deployed to examine sex ratios at all stages of gametocyte development *in vitro*. Improved differential staining and enhanced enrichment of gametocytes permitted robust estimation of the sex ratio in 3D7 cultures, and thus provide a basis for *in vitro* evaluation of drug effects on sex ratio, and so parasite transmission potential, in future studies.

## Materials and Methods

### Parasite culture

Parasites were cultured from the *P*. *falciparum* cell line 3D7A [22] (MRA-151; MR4-Malaria Research and Reference Reagent Resource Centre, Manassas, VA, USA) using the standard methods with slight modifications [7], [23], [24]. Parasites were maintained in T75 cell culture flasks (Iwaki, Japan) containing AB+ erythrocytes, and RPMI medium (PAA Laboratories, UK) supplemented with 10% AB serum. Cultures were incubated at 37°C and gassed for 1 minute every day (3% O_2_, 4% CO_2_, N_2_; BOC). Parasites were kept between 0.1–15% parasitemia at a haematocrit of 2–5%.

### Parasite harvesting

Magnetic activated cell sorting (MACS®; Milentyi BioTec, Bergisch Gladbach, Germany) [25], [26] was used for the purification of the parasites as previously described, with some modifications [24], [27]. Gametocytes were harvested on days 3 (stage II), 5 (stage III), 7 (stage IV) and 11 (stage V). Stages were identified after Field and Shute [28]. Before harvest, parasite cultures were washed twice with pre-warmed incomplete media (RPMI 1640 containing 25 mM HEPES and L-Glutamine, Gibco) at 500×g for 5 minutes and the supernatant was removed. MACS® columns (25CS columns, Miltenyi Biotec, Germany) were preheated in the incubator and filled with incomplete medium at 37°C. The pellet was diluted with incomplete medium at approximately 50% haematocrit, with slight adjustment for percentage parasitaemia. The parasites were then resuspended in pre-warmed RPMI and parasites were transferred into the column with a 1 ml pipette, until the whole sample moved through the column. Warm incomplete medium was added to the column until no RBCs were visible, to wash the column free of any remaining gametocytes. The eluate was then centrifuged at 500×g for 4 minutes at a minimum of 25°C and the supernatant was removed. Following this, gametocytes were washed and resuspended in a small volume of incomplete medium. To achieve the desired parasite density, a thin film was prepared on a glass slide and analyzed under a light microscope (magnified ×1000). Parasite density was then adjusted, by addition of incomplete medium, to the optimal of approximately 100 parasites per field of view and gametocytes were quantified exactly by haemocytometer (C-Chip, Neubauer, Germany).

For the observation of activated gametocytes, prior to slide preparation the gametocytes were incubated at room temperature for 10 minutes with 5× pellet volumes of cold incomplete medium including 20 µl of 100 µM xanthurenic acid (Sigma-Aldrich). This was not adjusted to reflect pellet volumes. Stage I to V and activated gametocytes were then pipetted onto multiwell slides (Hendley Essex), slides were air dried and stored at −20°C in a sealed box containing silica gel (Sigma-Aldrich) for long term storage.

### Isolation of gametocyte RNA

Parasites were harvested, purified and quantified with a haemocytometer (C-Chip, Neubauer, Germany) then added to 1.5 ml of Tri-Reagent® (Sigma, UK) pre-warmed at 37°C. Samples were gently shaken, left at room temperature for at least five minutes and were then stored at −80°C. Isolation of RNA was achieved by following the Tri-Reagent® manufacturer's protocol. Briefly, samples were thawed at 37°C and gently vortexed; 0.1 ml of 1-Bromo-3-chloropropane (Sigma-Aldrich) was added per 0.75 ml of Tri-Reagent®, vortexed and left at room temperature for 5 minutes. Samples were centrifuged for 30 minutes at 12,000×g at 4°C, which allowed the phase separation of the RNA and DNA. The upper aqueous phase containing the RNA was carefully removed, to which 0.5 ml of isopropanol (Sigma Aldrich) was added, allowing precipitation of the RNA. Samples were stored at 4°C overnight and then centrifuged at 12,000×g for 30 minutes. The RNA pellet was washed once in 75% ethanol (Sigma Aldrich), and the supernatant removed. Pellets were air-dried until no ethanol remained and resuspended in 20 µl of nuclease free water (Promega). The RNA solution was then stored at −80°C for up to three months.

### Obtaining cDNA from gametocyte RNA

20 µl of RNA dissolved in nuclease free water was added to a reagent mix containing 3 µl of 25 µg/µl random hexamer primers (Promega), 3 µl of 5 mM dNTPs (Promega), 3units of RQ-DNase (Invitrogen), 20 units of the restriction enzyme *Rsa*I (Invitrogen), 12 µl of 5× first strand RT-buffer (Invitrogen), 3 µl of 0.1 M DTT (Invitrogen) and nuclease free water to a total of 60 µl. The DNase/*Rsa*I reaction was incubated at 37°C for 1 hour, 94°C for 6 minutes and 12°C for 2 minutes. Each DNA-free RNA preparation was then split into two, one portion containing 50 µl, to which 320 units of reverse transcriptase (Superscript II; Invitrogen) were added for synthesis of cDNA, the other portion of 10 µl to which 0.33 µl nuclease free water (Promega) was added. All samples were incubated at 50°C for 1 hour and 70°C for 15 min; both reactions were then stored at −20°C.

### RT-PCR targeting Pfs16, Pfs25, Pfg377 and α-tubulin II

cDNA was prepared from parasites harvested on days 1, 3, 5, 7 and 11 of a gametocyte culture. The master mix for the RT-PCR was made with the following per 25 µl reaction: 2.5 µl of 10× NH_4_ buffer (Bioline), 0.5 µl of 50 mM MgCl_2_ (Bioline), 0.5 µl of 5 µM dNTPs (Promega), 1 µl of 5 µM of the chosen forward and reverse primers, 25 units of *Taq* polymerase (Bioline) and 3–5 µl of cDNA. Nuclease free water (Promega) was used to bring the reaction to a volume of 25 µl. Positive controls, containing 3D7 DNA and negative controls containing nuclease free water (Promega) were included in each run. Amplification primers used were:

α-tubulin II: forward (SA05) 5′-TGAACATGGAATTCAACCGG-3′

reverse (SA06) 5′- CGTCAACGACGGTGGGTTC-3′

Pfs16: forward (SA07) 5′-TTCTTCGCTTTTGCAAACCT-3′

reverse (SA08) 5′-AAAGGCATTTTGTCAGCAGAA-3′

Pfg377: forward (SA09) 5′-CCCCATTTCCTCCTAAAGTACC-3′

reverse (SA10) 5′-CTGGTTCTGCTTCTGGTTCC-3′

The cycling conditions for α-tubulin II (SA05+SA06) were 95°C for 6 minutes, 95°C for 5 seconds, 58°C for 1 minute, 70°C for 30 seconds (40 cycles). The cycling conditions for Pfs16 (SA07+SA08) were 94°C for 6 minutes, 94°C for 5 seconds, 53°C for 1 minute, 70°C for 30 seconds (40 cycles). The cycling conditions for Pfg377 (SA09–SA10) were 96°C for 6 minutes, 96°C for 5 seconds, 59°C for 1 minute, 70°C for 30 seconds (40 cycles).

cDNA and DNA amplification products were fractionated on 1.2% (0.5× TBE) agarose gels (Sigma-Aldrich), containing 2.5 µl of ethidium bromide (10 mg/l) at 100 V for 1 to 1.5 hours, and visualised over UV light.

### Sex discrimination in Giemsa-stained gametocytes

Asexual parasites and gametocytes were monitored daily by preparing thin films of approximately 10 µl of the culture at 50% haematocrit. Preparations were methanol fixed for 10 seconds and stained in 10% Improved R66 Giemsa (BDH) in phosphate buffer (pH 7.4), applied with a syringe containing a 0.45 µm pore size filter (Millex) to remove particulate matter. The preparations were examined at ×1000 magnification and the sex of the mature gametocytes was identified based on the five characters described by Carter *et al*. [7]. The sex ratio was then calculated by dividing the total number of male gametocytes observed, by the total number of male and female gametocytes observed. This denominator comprised all gametocytes where at least 4 out of the 5 discriminatory characters could be identified; the number of undetermined parasites was also noted.

### Immuno Fluorescent Antibody Test

After considerable trouble-shooting to obtain reliable double-staining results consistent between experiments, we adopted the following modification of standard protocols. Slides were removed from the freezer, air dried in a ‘dry box’ containing silica gel for 15 minutes, and then fixed in ice cold anhydrous acetone (BDH) for 30 minutes. The slides were washed with PBS (Sigma Aldrich) and excess fluid between the wells removed. PBS-1% BSA, 0.1% Tween®20 was added to each well to prevent non-specific binding of the antibodies. Slides remained for 30 minutes in a humid sealed box, containing moist paper towels to prevent the wells from drying out.

Slides were washed in PBS and the primary polyclonal antibody for α-tubulin II (1∶7000 dilution in PBS-1% BSA, 0.1% Tween®20) was added to the wells and incubated at room temperature for 1 hour. The slides were washed with PBS and the fluorescent secondary antibody (1∶400 dilution in PBS) rhodamine-conjugated donkey anti-rabbit IgG (Jackson Immuno Research) was added for 30 minutes. The slide was washed again and the second primary monoclonal antibody Pfg377 (1∶400 dilution in PBS-1% BSA) was added for 1 hour. Slides were washed and the fluoroscein-conjugated secondary antibody donkey anti-rat IgG (Jackson Immuno Research) was added for 45 minutes. Antibodies were added sequentially, due to the differential sensitivity and incubation time needed. Primary and secondary antibodies were incubated at room temperature.

Following antibody incubations, the slides were washed with PBS and 40 µl of Vectashield® containing DAPI (Vector Laboratories) was added to the slide, a coverslip was mounted and slides were sealed with nail varnish and stored at 4°C for a maximum of 2 days until visualized and photographed by confocal microscopy (Zeiss Axioplan LSM510). Parasites were magnified ×1000 under the ×100 immersion oil objective, and fluorescent signals detected at 488 nm for fluoroscein (Pfg377-female gametocytes), 543 nm for rhodamine (α-tubulin II-male gametocytes), and 403 nm for DAPI (staining the nuclear material), using Zeiss LSM software. The anti-Pfg377 antibody (anti-rat) was a kind gift of Pietro Alano. Anti-α-tubulin II (anti-rabbit) was obtained from the MR4 (MRA-37 MR4, Manassas, VA.).

### Application of Image ProPlus 6.3 for sexing gametocytes using IFAT

IFAT images were analyzed with the Image-ProPlus 6.3 software, which allowed tagging individual gametocytes according to stage and colour. Parasites staining only red (α-tubulin II) were tagged as males, whereas parasites staining with red and green (Pfg377 and α-tubulin II) were tagged as females. Gametocytes that only stained with DAPI were also noted.

## Results

### Sex-specific mRNA in preparations of developing gametocytes

**Figure 1:**
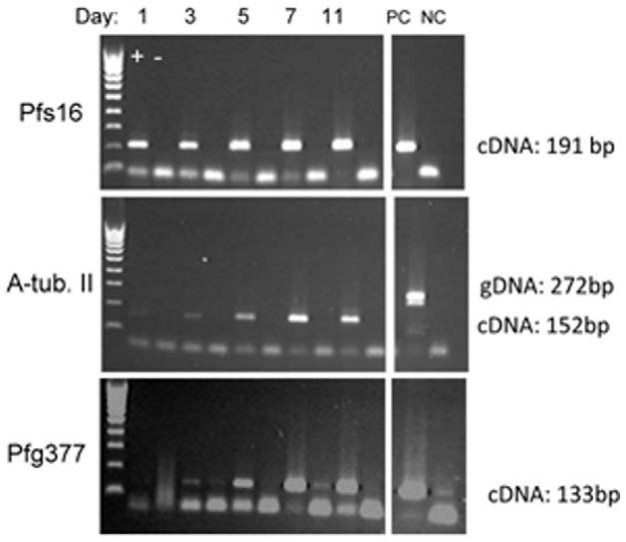
RT-PCR of sexual stage and sex specific proteins during gametocytogenesis. Transcripts of Pfs16, α-tubulin II and Pfg377 were amplified from preparations of stage I–V gametocytes. Gel electrophoresis of amplified products; + and − refer to presence or absence of reverse-transcriptase in the cDNA reaction prior to amplification. PC: positive control; NC: negative control. Cultures were not 100% synchronous. All samples (including positive controls) were run on a single gel at the same time. Lower bands (<100 bp) in each panel, particularly prominent in the absence of cDNA amplification, are primer dimers. Minor contamination of pfg377 DNA is visible in lanes 4, 8 and NC, lower panel.

RT-PCR of mRNA from stage-specific preparations of synchronised developing gametocytes demonstrated that *α-tubII* transcripts were abundant throughout development (Fig. 1, 2^nd^ panel), whereas *pfg377* transcripts were barely detectable in stage I/IIa gametocytes, becoming very abundant in later stage preparations (Fig. 1, 3^rd^ panel). Transcripts of *pfs16* were abundant throughout gametocyte development as expected from previous studies [29], confirming the integrity and specificity of the RNA preparations. This lack of coordination between the two genes of interest suggests that quantitative measurements of *α-tubII* and *pfg377* transcript accumulation during development would not provide stable estimates of sex ratio; other transcripts may be more suited to this purpose.

### Sex ratios established using light microscopy

**Figure 2:**
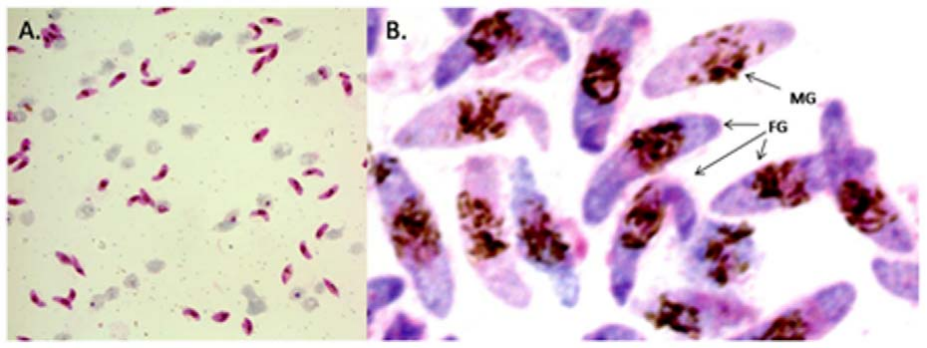
Sex ratios established using light microscopy. Purified stage V gametocytes stained with Giemsa. A.) 3D7 magnet-purified gametocytes magnified ×1000. B.) (Detail) The cytoplasm of male gametocytes (MG) can be seen to stain pink and that of female gametocytes (FG) to stain purple; male gametocytes are smaller than females, the nucleus is bigger in males than in females, the granules of the malaria pigment are centrally located in female gametocytes and more widely scattered in males gametocytes The other 4 discriminatory characters can also be discerned.

Giemsa-stained stage V (day 11) purified parasites were sexed based on the five characters of Carter *et al*. [7]. Magnetic purification ensured high densities of mature gametocytes on a single slide which facilitated sex ratio quantification (Fig. 2). Three independent gametocyte cultures were evaluated, contributing 110, 114 and 116 mature gametocytes respectively, with 92–96% of these able to be scored for 4 of the 5 characters. *P*. *falciparum* clone 3D7 was confirmed as female biased, having sex ratios of 0.140 (S.E. 0.009), 0.125 (S.E. 0.008) and 0.120 (S.E. 0.008), respectively, in three separate cultures, giving an average estimate of gametocyte sex ratio by light microscopy of 0.128, which approximates to seven females per male gametocyte.

### Pfg377 and α-tubulin II expression during gametocyte development

**Figure 3:**
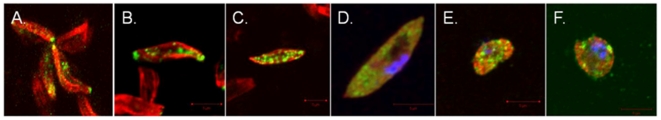
Stage III and later gametocytes visualized with anti-Pfg377 and anti-α-tubulin II antibodies. Fluorescent staining of α-tubuIin (red) generated a characteristic striated pattern, particularly in earlier gametocytes. Expression of Pfg377 (green) was not seen prior to stage III. A) stage III; B) late stage III; C) stage IV; D) late stage IV; E) stage V; F) activated female gametocyte. Nuclear material was stained with DAPI, appearing blue in colour. Parasites were magnified ×1000.

Evidence of Pfg377 expression was observed from early stage III gametocytes onwards (day 4–5). In these immature gametocytes, Pfg377 antibodies recognised granular clusters at the tips of the gametocytes and in the centre of the cell (figure 3A, 3B). From stage IV onwards, staining generated by Pfg377 antibodies remained punctuate but was found throughout each parasite, suggesting that the protein was more homogenously distributed in later stages (Fig. 3C, 3D). Once the gametocytes were activated, Pfg377 clusters reappeared near the circumference of the parasitophorous vacuole in some gametocytes (Fig. 3E, 3F), as previously reported by Severini et al. [13].

**Figure 4:**
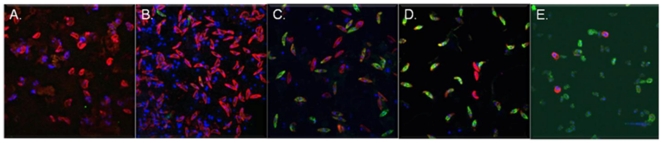
Differential staining of gametocytes during gametocytogenesis. Pfg377 and α-tubulin II double-stained IFAT slides prepared on: A) day 2 (stage II); B) day 5 (stage III); C) day 7 (stage IV); D) day 11 (stage V); E) after gametocyte activation (AG). Parasites were magnified ×1000. Early stage parasites were more likely to be damaged during the purification process, and appear as stained with DAPI alone (panels A, B).

Expression of α-tubulin II was detected as early as stage I, the fluorescent signal appearing to increase in intensity in a subset of (presumably male) gametocytes as they matured (Fig. 4A–E). From stage II to III, α -tubulin II was found to be concentrated longitudinally at the edges of the parasite, and striations were seen throughout the cell (Fig. 3A, B, C). From stage I to III, α -tubulin II fluorescent intensity was similar among all gametocytes at the same stage of maturity, yet stage IV and stage V gametocytes that reacted with anti-Pfg377 revealed much lower expression levels of α-tubulin II than those gametocytes not expressing Pfg377 (Fig. 4C, D, E). Particularly striking was the strong fluorescence in activated gametocytes that only reacted with anti-α-tubulin II antibodies and not Pfg377 antibodies (presumably male), whereas the activated gametocytes also reacting with Pfg377 (presumably female) showed very low expression levels of α-tubulin II (Fig. 4E).

These antibody-staining results suggest discrimination between fully mature and/or activated male and female gametocytes, prior to induction of ex-flagellation and female gamete emergence, can be readily achieved with this procedure. Discrimination can be made on the basis that Pfg377 staining, together with a reduced anti-α-tubulin II signal, is characteristic of female parasites in the later stages of development. Reliable specific staining of emergent microgametes was not achieved.

Discrimination between the males and females was not possible at stages I–III of gametocyte development, when all gametocytes stain strongly with anti-α-tubulin II antibodies, and females do not express sufficient Pfg377 protein to produce a definitive pattern (Fig. 4 A–D). Counting of the gametocytes recognised by one or both antibodies clearly demonstrate that changes in relative expression of both proteins occur during development (Table 1). In stages II and III, 55% and 43% of gametocytes, respectively, did not react with either of the antibodies, partly due to the presence of damaged DAPI-stained parasites. After activation, only 12% of the gametocytes were not recognised by either antibody. The proportion staining with anti-PfG377 antibodies increased dramatically in stages IV and V, indicating a likely female-bias of the sex ratio, which was obscured earlier in development by the lack of Pfg377 expression. These findings are in broad agreement with the pattern of abundance of the two transcripts encoding these two proteins during development (Fig. 1).

**Table 1:** ‘Apparent’ sex ratio during gametocytogenesis.

Stage	II	III	IV	V	AG
DAPI	129	611	207	1916	350
% sexed	45.0	57.1	72.9	81.7	88.6
Alapha-tub.II +	58	342	26	221	12
Pfg377/A.tub. II +	0	7	125	1344	298
% Alpha-tub. II +	45	56	13	12	3
% Pfg377 +	0	1.1	60.4	70.15	85.1
Sex ratio	1	0.980	0.17	0.128	0.039
Standard error	0	0.011	0.06	0.056	0.021

IFAT counts were performed on gametocyte preparations of stage II, stage III, stage IV, stage V, and 30 minutes after induction of activation (AG). Standard error is estimated for the ratio based on error calculated for the mean estimates of the proportion of males across the three independent cultures analysed.

% Alpha-tub. II+, % Pfg377+: indicate the percentage of gametocytes reacting with the given α-tubulin II and Pfg377 antibodies respectively.

### Distinguishing mature male and female mature gametocytes using IFAT

**Figure 5:**
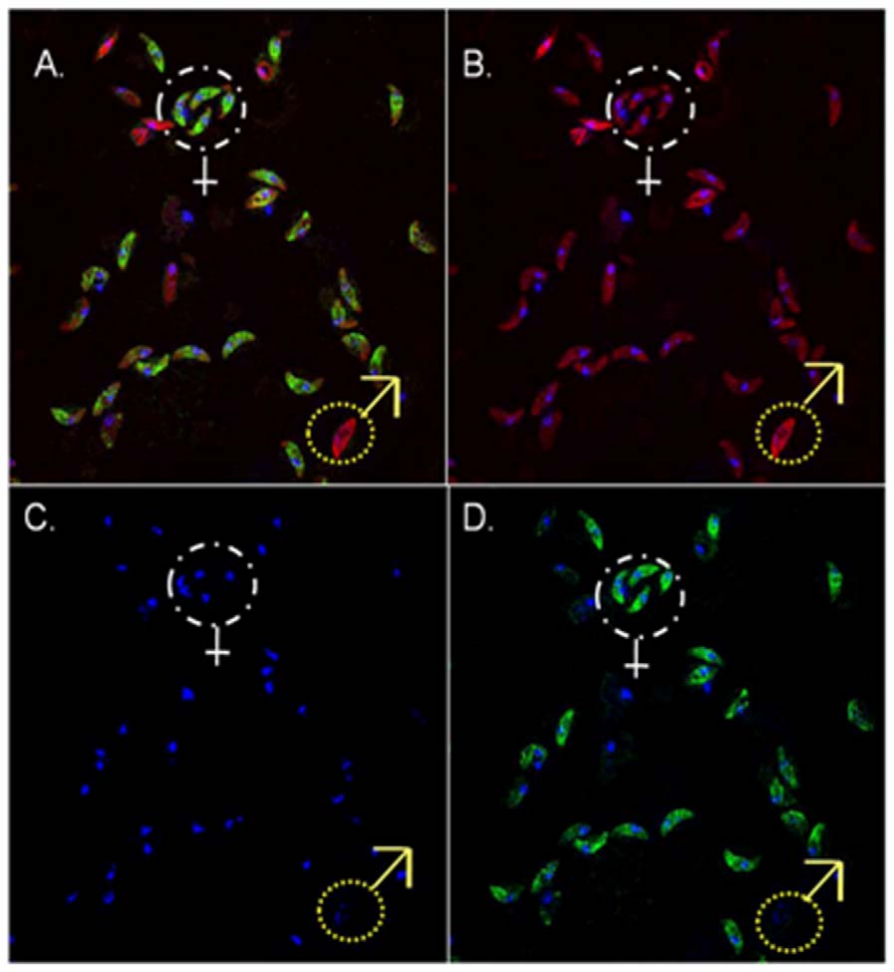
Distinguishing between male and female gametocytes. A) IFAT image of stage V gametocytes showing all fluorophores from the secondary antibodies and DAPI (red, green and blue). B) Rhodamine (red) is visualized, which reacts with anti-α-tubulin II antibodies. C) DAPI (blue) is visualized, reacting with the nuclear material. D) FITC (green) is visualized, reacting with anti-Pfg377 antibodies. The white dotted circle shows gametocytes staining with anti-Pfg377 and anti-α-tubulin II (females), whereas the yellow dotted circle reveals one gametocyte that only reacts with anti-α-tubulin II antibodies (male). It can be seen that gametocytes which reacted with anti-Pfg377 antibodies, also reacted with anti-α-tubulin II antibodies, when comparing B) and D). Parasites were magnified ×1000.

Based on the results shown in Figures 3 and 4, sex ratios were estimated in preparations of late stage IV and stage V (non-activated) gametocytes. The primary marker used was Pfg377, assumed to be a female-specific protein, and anti-α-tubulin II, which reacts with both sexes, albeit at different intensities, was taken as a secondary marker to provide a denominator of viable, intact gametocytes of both sexes. The use of a secondary marker in addition to DAPI nuclear staining prevents over-estimation of the total number of gametocytes, caused by nuclear remnants from gametocytes damaged in the preparation process (Fig. 4B). On this basis, sex ratios were estimated from preparations of mature, non-activated gametocytes (Fig. 5), in three independent cultures, two of which were among those evaluated by light microscopy. These estimates were 0.108 (S.E. 0.041), 0.210 (S.E. 0.118) and 0.119 (S.E. 0.041), and were thus in agreement with the female-biased estimates of sex ratio obtained by light microscopy.

## Discussion

In this study, systematic stage-specific preparations of *P*. *falciparum* gametocytes developing *in vitro* were examined by IFAT for expression of two proteins, α-*tubulin* II and PfG377, previously used as male- and female-specific gametocyte markers, respectively. We show, in contrast to previous studies, that α-tubulin II is expressed by both sexes of *P*. *falciparum* gametocytes, but that from stage IV onwards was markedly reduced in abundance in female gametocytes. This is consistent with studies of the rodent parasite *P*. *berghei*, demonstrating α-tubulin II expression in both sexes [19], [20]. With Pfg377 as a primary marker recognising only female gametocytes, and α-tubulin II as a secondary marker for accurately enumerating the denominator of viable gametocytes, robust sex ratios could be derived for preparations of gametocytes at stage IV or later. These estimates agreed with the sex ratio obtained by light microscopic examination of Giemsa-stained mature 3D7a gametocyte preparations, and are similar to those obtained by others for this parasite clone [14]. However, following activation by xanthurenic acid, rapid changes in protein localisation and abundance occurred, and stable sex ratios could no longer be derived from IFAT studies. RNA-based studies demonstrated that *pfg377* and α*-tubII* transcripts did not accumulate in a coordinated fashion during development. This suggests that quantitative cDNA amplification methods for estimating sex ratios with these two markers would be affected by even quite small differences in gametocyte maturity among the preparations being compared, as maturity is expected to confound relative transcript abundance prior to stage V.

Three different functional classes of microtubules have been identified in *Plasmodium*; axonemal and flagellar microtubules, involved in the movement of the male gamete; subpellicular microtubules, involved in the cell structure and motility, and spindle – associated microtubules, which play a role in cell division [30]. Scanning electron microscopy (SEM) and IFAT images have reported that male gametocytes contain all classes of microtubules, whereas female gametocytes have been reported to have only subpellicular microtubules [8]. After emergence from the erythrocyte, female gametes do not contain any microtubules whereas male gametes are reported to contain only axonemal microtubules [8], [30]. In *Plasmodium*, two α-tubulins, I and II, which share 95% sequence identity, and one β-tubulin are present [31]–[33]. Pfg377 is confined to *P*. *falciparum* female gametocytes, the only protein that has been associated with osmiophilic bodies, intracellular membrane-bound vesicles found beneath the subpellicular membrane from stage III gametocytes onwards [13]. In clones of *P*. *falciparum* 3D7 with the *pfg377* gene knocked out, female gamete emergence was severely impaired, and infectiousness of gametocytes to mosquitoes greatly reduced, although not entirely ablated [16].

The results of our IFAT studies are broadly consistent with the known biology of α-tubulin II and Pfg377, described in the preceding paragraph, but do show for the first time that the former is not a male-specific protein in *P*. *falciparum*, in contrast to the findings of Rawlings *et al*. [12]. Of additional significance is the lack of expression of Pfg377 transcripts (Fig. 1) and protein (Fig. 4) prior to Stage IV of gametocyte development. Thus any technique which estimated sex ratio based on Pfg377 transcript or protein abundance would deliver an “apparent” sex ratio that was extremely male biased prior to stage IV. As commitment of any particular *P*. *falciparum* parasite to male or female gametocyte development occurs in the parental asexual parasite (Bruce *et al*., 1990), this apparent ratio is an artefact of the markers chosen, as α-tubulin II is expressed by both sexes in these early stages, and female gametocytes do not produce reliably detectable levels of Pfg377 until they reach stage IV. Further, any contamination of mature gametocyte preparations with earlier stages, due to inadequate synchronisation, will also produce artefactually male-biased sex ratio estimates. The development of antibody-based fluorescent staining protocols for male- and female-specific proteins expressed earlier in gametocyte development would overcome this problem. Drew and Reece [34] developed a qRT-PCR assay for the quantification of sex ratios in *P*. *chabaudi*, however the extended maturation of *P*. *falciparum* gametocytes over many days means the relative abundance of sex-specific mRNA through development must be characterised prior to calibration of such assays against microscopic data. Indeed, it may be necessary to quantify transcript abundance of more than one male- and female-specific gene in order to correct for maturity in estimating sex-ratio. This important effect of maturity is unique to gametocyte development in *P*. *falciparum*, among the malaria parasite species that can be studied in the laboratory.

The ability to rapidly and accurately derive estimates of gametocyte sex ratio provides a valuable additional phenotype for studies of the *in vitro* effect of antimalarial compounds on parasite transmission potential. Enumeration of gametocytes *in toto* may fail to recognise important transmission-blocking effects which only affect one sex, particularly if these were male gametocytes; loss of all males would completely prevent transmission to mosquitoes, but cause only a small decrease in total gametocyte numbers if females were unaffected. The ideal method for routine evaluation of drug effects is unlikely to be IFAT, as it is difficult to scale-up for high-throughput assays, but our antibody-based methods could be adapted to flow cytometrics. Further, we suggest that the approach we have described is ideal for comparative studies of different gametocyte-producing lines, phenotypic studies of transgenic gametocyte lines following targeted gene-disruption, and, most importantly, for validation of high-throughput methods such as quantitative reverse-transcriptase PCR sex ratio assays, currently in development for *P*. *falciparum* in our laboratory.
